# Antimicrobial Polymer Films with Grape Seed and Skin Extracts for Food Packaging

**DOI:** 10.3390/microorganisms12071378

**Published:** 2024-07-06

**Authors:** Yavor Ivanov, Tzonka Godjevargova

**Affiliations:** Department Biotechnology, University “prof. d-r A. Zlatarov”, 8010 Burgas, Bulgaria; qvor_burgas@abv.bg

**Keywords:** grape seed extract, grape skin extract, preparation, antioxidant, antimicrobial activity, antimicrobial packaging, intelligent packaging

## Abstract

The development of antimicrobial food packaging is a very important and current goal, but it still difficult to implement in practice. Reducing microbial contamination and preserving food quality are very important tasks for food manufacturers as the use of antimicrobial packaging can preserve the health of consumers. On the other hand, the difficulty of degrading packaging materials, leading to environmental pollution, is also an important problem. These problems can be solved by using biodegradable biopolymers and antimicrobial agents in the production of food packaging. Very suitable antimicrobial agents are grape seed and skin extracts as they have high antioxidant and antimicrobial capacity and are obtained from grape pomace, a waste product of winemaking. The present review presents the valuable bioactive compounds contained in grape seeds and skins, the methods used to obtain the extracts, and their antimicrobial and antioxidant properties. Then, the application of grape seed and skin extracts for the production of antimicrobial packaging is reviewed. Emphasis is placed on antimicrobial packaging based on various biopolymers. Special attention is also paid to the application of the extract of grape skins to obtain intelligent indicator packages for the continuous monitoring of the freshness and quality of foods. The focus is mainly placed on the antimicrobial properties of the packaging against different types of microorganisms and their applications for food packaging. The presented data prove the good potential of grape seed and skin extracts to be used as active agents in the preparation of antimicrobial food packaging.

## 1. Introduction

Food contamination by pathogenic microorganisms is a serious threat to food safety and public health. Microorganisms that enter food can cause the formation of toxic compounds and change the quality of food [1,2]. Preserving the quality of food and increasing its shelf life can be successfully achieved by using antimicrobial packaging containing biologically active substances with antioxidant and antimicrobial activity [3,4]. The use of this type of packaging not only prevents microbial contamination and proliferation, but also leads to a reduction in the direct addition of synthetic preservatives in food, as they have adverse effects on human health [5,6]. In recent years, the food industry has widely used phenolic compounds from natural plant sources as antimicrobial and antioxidant agents. The latter are recognized as safe materials by food regulatory agencies, such as the Food and Drug Administration (FDA). These phenolic compounds possess unique antimutagenic, anticarcinogenic, antifungal, insecticidal, bactericidal, and antiviral actions [7,8]. They are not only used as ingredients in food packaging, but also as alternatives to synthetic preservatives added directly to food [7,9].

Polyphenols are compounds with high antioxidant and antimicrobial activity. These are found naturally in a number of plants—including herbs, fruits, and greens. Grapes have a very high content of polyphenols. The structural variation in phenolic compounds alters their antimicrobial activity. In recent years, there has been a growing demand for the recovery of bioactive substances from a large number of fruit and vegetable wastes [10]. The wine industry discards a large amount of grape pomace, consisting mainly of skins, seeds, and raisins [11,12]. The use of grape pomace or the seeds and skins separated from it to obtain a bioproduct with a high added value has certain advantages over other types of fruit and vegetable wastes. First of all, it is an affordable raw material, and about 9 million tons of pomace are thrown away from the wine production process throughout the world every year [12]. Similar large amounts of waste are thrown away during the processing of lemons, oranges, tomatoes, apples, etc. The polyphenolic composition of the extracts from grape seeds, skins, and pomace differs from that of the rest of the waste. They mainly contain phenolic acids, flavonoids, anthocyanins, procyanidins, and flavanols [11,12,13]. The contents of procyanidins, epicatechin, and catechin in grape seed extracts are very high, and the content of anthocyanins in grape skin extracts is also very high. For comparison, in the extracts from lemon, orange, and tomato waste, phenolic acids predominate and procyanidins are absent [10]. The indicated polyphenols extracted from grape seeds and skins have high antioxidant, antimicrobial, and antilipid capacities, making them very applicable in the food industry, medicine, cosmetics, etc. One of the most important goals regarding the application of these valuable bioproducts is obtaining antimicrobial food packaging.

The inclusion of antimicrobial agents in food packaging is carried out by pre-preparing a polymer composition, followed by the formation of a film [14]. Different polymers are used—synthetic polymers from petroleum products and biodegradable edible polymers [15]. In recent years, biodegradable packaging containing antimicrobial biologically active substances has become increasingly popular [16]. There is also great interest in smart biodegradable packaging, which, thanks to the included antimicrobial and antioxidant agents, changes color and shows the packaged food’s degree of possible microbial contamination over time [17]. Many publications have convincingly demonstrated the efficient inhibition of microbial contamination in foods using antimicrobial packaging containing polyphenols [18,19] and convincingly proved the relevance of research in this direction. There are also publications describing the successful application of grape seed and skin extracts to obtain antimicrobial food packaging [20,21], but there is no comprehensive review uniting the available information on these products. Utilizing waste from the wine industry and obtaining valuable, economically viable bioproducts with high antioxidant and antimicrobial activities solve two very important tasks for society, namely the protection of the environment and human health.

This review will discuss in detail the topic of obtaining grape seed and skin extracts from wine industry waste, proving their antioxidant and antimicrobial capacities and the efficacy of their application to obtain antimicrobial food packaging.

## 2. Polyphenols in Grape Seed and Skin Extracts and Methods for Preparing Extracts

Large amounts of grape pomace are thrown away as waste during wine production. It contains the seeds, pulp, skins, and stalks of the fruit. The contents of seeds and skins are the highest in grape pomace. The total amount of extractable phenols in the seeds of different grape varieties is known to vary from 60 to 70%, that in the skins is in the range of 28–35%, and that in the pulp is about 10% [22]. These facts clearly indicate that grape seeds and skins are a valuable source of phenolic compounds that can be extracted from grape seeds and skins separated from waste grape pomace.

The main polyphenols contained in grapes are proanthocyanidins, anthocyanins, flavonols, flavanols, resveratrols, and phenolic acids [22,23,24,25,26,27]. The phenolic compounds in different parts of grapes (skins and seeds) are different (Table 1).

Flavonoids are widely distributed in seeds and skins and mainly contain (+)-catechins, (−)-epicatechin, and procyanidin polymers. The gallic acid and syringic acid are contained in a high percentage both in grape seeds and in grape skins. Proanthocyanidins are also found in a high concentration in grape seeds and skins [23]. Anthocyanins are pigments and mainly exist in red grape skins. Flavonols (myricetin, quercetin, kaempferol, and trans-resveratrol) are mostly found in red grape skins [24,26,27].

**Figure 1:**
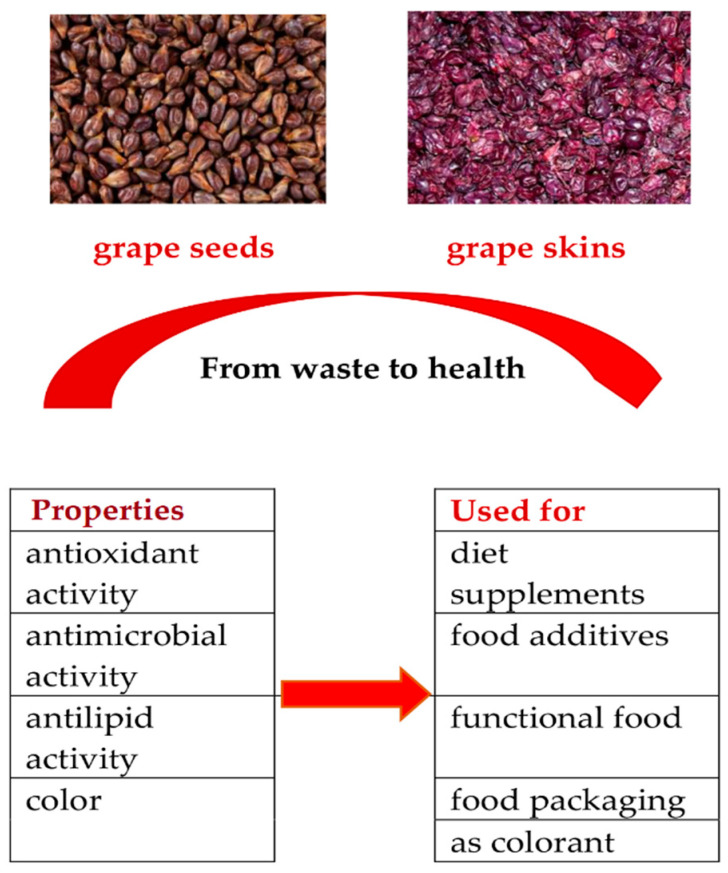
The properties and utilization of grape seed and skin extracts in the food industry.

These biologically active substances have antimicrobial, antioxidant, and antilipid properties. They have various practical applications; for example, they can be used as diet supplements, food additives, functional foods, and colorants, as shown in Figure 1. These bioproducts can be applied directly to foods as natural preservatives and/or be included in food packaging in order to preserve food quality [3,4,7,9].

To obtain quality extracts from grape seeds and skins with high antioxidant and antimicrobial capacities and with a high yield, the methods used for carrying out extraction are of essential importance. Conventional and unconventional extraction methods are used [29,30]. One of the conventional methods, the most commonly used technique for isolating phenolic compounds from plant material, is solid–liquid extraction (SLE), [22,29]. The following solvents are used to conduct SLE: ethanol, methanol, acetone, diethyl ether, and ethyl acetate mixed with water in different ratios. Ethanol is non-toxic, and only this solvent has been recognized as safe by the European Food Safety Authority and the FAO/WHO Expert Committee on Food Additives. This method allows for the very good extraction of phenolic compounds from grapes, but the use of large amounts of organic solvents poses health risks and is unfavorable for the environment. Several improved non-conventional methods for extracting phenols from grapes have been developed, such as supercritical fluid (SFE), pressurized liquid (PLE), ultrasound-assisted (UAE), and microwave-assisted (MAE) extraction [31,32]. These extraction methods significantly reduce the consumption of solvents and increase the extraction rate by simplifying the process. Supercritical fluid extraction (SFE) is an effective technique that is widely applied for the isolation of polyphenols from grapes [33]. The advantages of this technique are the achievement of faster and more efficient extraction due to the very high solvent capacity and the distinctive physicochemical properties of supercritical fluids. (CO_2_). When using supercritical fluids [31,33], extraction is achieved in ten minutes, while conventional solid–liquid extraction lasts several hours or even days. In some cases, supercritical fluid is modified with co-solvents in order to improve its polarity [34,35]. The extraction of resveratrol from grape skin has also been optimized by adding ethanol to the CO_2_ supercritical fluid [36]. Chafer et al. conducted SFE of polyphenols from five varieties of grape skins and found that the most suitable conditions are 60 °C, 250 bar, and 20% ethanol as a CO_2_ modifier [37]. In various studies, SFE has been shown to be selective for phenols, such as gallic acid, catechin, epicatechin, and quercetin, and high yields of these polyphenols from grape pomace have been provided [33,38]. MAE also offers lower solvent consumption, reduced waste release to the environment, faster extraction (typically 1–20 min), and higher energy savings because microwaves only heat the sample (not the apparatus) [39,40,41,42,43,44]. Some authors recommend UAE and indicate that it allows for the preparation of polyphenol-rich grape extract with high antioxidant activity [45,46,47]. PLE has been widely used for the extraction of antioxidant compounds from wine industry wastes [48,49]. PLE is based on the use of conventional liquid solvents under subcritical conditions with controlled temperature and pressure. Like all non-conventional extraction methods, PLE uses less solvent, and extraction takes place in less time. In recent years, there has been considerable interest in using the by-products of the wine industry for the production of antioxidants using high-pressure techniques. This interest has been discussed by Otero-Pareja et al. [50].

There are many publications comparing the merits of individual extraction methods. Pinheiro et al. compared the extraction of catechin and epicatechin from tea leaves and grape seeds obtained using ultrasound (UAE) and PLE [51]. Other comparative studies have shown that in the digestion of waste from the wine industry, PLE is more efficient than conventional solvent extraction, MAE and UAE, and high levels of phenolics are obtained from grape pomace and grape skin [52,53]. Casazza et al. [31] compared the use of four types of extraction (SLE, ultrasound-assisted extraction (UAE), microwave-assisted extraction (MAE), and high pressure and temperature extraction (HPTE)) and their impact on the yield and antioxidant capacity of grape seed and skin extracts. HPTE and MAE were found to be more efficient than the other two methods. Some authors pointed out that PLE and SFE are preferable to MAE because they can be applied to extract biologically active substances from different quantities of raw materials [54,55]. PLE and SFE can be applied in laboratory-scale, pilot plant-scale, and industrial-scale (tons of feedstock) systems. On the other hand, several studies have demonstrated the economic viability of SFE and PLE for the extraction of phenols from winery waste [54] and from other raw materials [55].

Although SFE, MAE, and UAE are considered green and interesting procedures for the extraction of bioactive compounds, the conventional SLE method is still used. It was found that unconventional techniques cannot be used to extract high-molecular-weight polyphenols such as proanthocyanidins. These compounds are more easily extracted by conventional extraction methods [31,32]. Other authors have carried out the optimization of the UAE [56] and MAE [44] methods and described that they obtain better results for the extraction of proanthocyanidins compared to conventional methods. Kinetic models have also been developed through which the optimal conditions for the extraction of polyphenols from grape seeds are specified very well [57]. Some studies have shown that the two techniques, SFE and PLE [12,44,58], are successfully used to obtain phenolic compounds from grape pomace in large quantities, but there are not enough comparative studies to evaluate the performance of the extracted anthocyanins and phenols from this raw material. It is difficult to make a real comparison of the individual polyphenol extraction techniques. In order to make such a judgment, it is important to analyze and compare more indicators of the obtained extracts—the yield, total phenolic content, total contents of anthocyanins and flavonoids, and antioxidant and antimicrobial activities.

## 3. Antioxidant and Antimicrobial Capacities of Grape Seed and Skin Extracts

Different grape varieties contain different phenolic compounds in different parts of the grape in terms of structure and quantity. In addition, the content of phenols in the individual parts of the grapes depends on the geographical location of the vineyards and the climate. This shows that in order to assess the biologically active properties of a given grape extract, an individual approach must be taken. The content and structure of phenolic compounds in grape extracts determine their antioxidant and antimicrobial properties. In order to qualify the important activities of each isolated grape extract, the following general parameters are determined: the total phenolic content (TPC), total flavonoids (TF), total anthocyanins (TA), and procyanidins (PC). The methods for their determination are described in many publications [59,60]. Table 2 presents the values of these parameters for some seed and skin extracts of different types of red and white grapes, which were published by some authors.

The values of TPC, TF, and PC in the white grape varieties are lower compared to those of red grapes. The reason for this is the fact that anthocyanins are not synthesized in the skins of white grapes (Table 2). Anthocyanins are the main polyphenolics in red grape skins [26,27,61]. The TPC, TF, TA, and PC values of the grape seed extracts were higher compared to the same ones from the grape skins [62,63,64,65]. The determination of the individual components in the extracts gives an even clearer idea of the quantitative and qualitative compositions of the grape extracts. The individual compositions of the extracts were determined by the HPLC method [66,67] and the application of gradient elution. In this analysis, the selection of elution solvents to achieve the separation of both polar and non-polar compounds in the extracts is essential. The results presented in Table 2 clearly show that the values of total phenolic compounds of grape seed and skin extracts, as determined by HPLC, are high. Many authors have proven that these values are the highest in grape seed extracts, followed by those in skins and pulp, and finally in stem extracts [68,69,70]. In order to fully characterize the extracts, an important parameter is their antioxidant capacity. There are different methods for determining the antioxidant capacity [71,72,73], but the most frequently used analyses are 1,1-diphenyl-2-picryhydrazyl (DPPT), 2,2′-azino-bis-(3-ethylbenzothiazoline-6-sulfonic acid (ABTS), oxygen radical absorbance capacity (ORAC), and Ferric Reducing Antioxidant Power (FRAP). Table 2 presents the values of these parameters for the enlisted grape varieties. A very good correlation between the values of the total phenolic parameters of the extracts and their antioxidant activity is evident. The antioxidant capacities of the red grape seed and skin extracts are higher than those of the white ones. These data convincingly show that grape seeds and skins are suitable sources for the production of valuable phenolic extracts and are economically and ecologically beneficial as waste from the wine industry is recovered.

**Table 2:** Polyphenolic content in skin and seed extracts of white and red grapes.

Grape Variety	Extract source	TPC, mg/g	TA, mg/g	TF, mg/g	PC, mg/g	DPPT, µmol TE/g	ABTS, µmol TE/g	References
Albarossa (red)	Skins	37.5 ± 0.6	21.5 ± 1.0	47.5 ± 1.3	24.1 ± 1.6	-	43.5 ± 2.4 *	[62]
	Seeds	73.7 ± 0.2	-	105.6 ± 0.7	87.4 ± 0.8		109.7 ± 6.8 *	
Barbera (red)	Skins	33.2 ± 0.8	17.1 ± 1.0	31.2 ± 1.5	16.6 ± 1.5	-	34.2 ± 1.5 *	
	Seeds	83.8 ± 7.6	-	128.6 ± 16.1	85.2 ± 3.3		117.6 ± 10.5 *	
Nebbiolo (red)	Skins	36.7 ± 1.7	9.4 ± 0.8	32.5 ± 1.3	43.2 ± 4.6	-	49.7 ± 4.4 *	
	Seeds	106.5 ± 9.0	-	162.8 ± 21.4	125.4 ± 10.9		184.9 ± 7.9 *	
Uvalino (red)	Skins	34.5 ± 2.9	12.4 ± 1.6	33.5 ± 3.3	30.5 ± 3.7	-	51.7 ± 4.9 *	
	Seeds	107.8 ± 0.2	-	158.4 ± 0.1	152.0 ± 2.5		185.5 ± 1.3 *	
Vranec (red)	Skins	48.3 ± 0.08	8.40 ± 1.13	10.2 ± 0.04	-	Skin extracts—from 15.7 to 113.3 Seed extracts—from 16.8 to 92	-	[63]
	Seeds	139 ± 0.48	-	52.0 ± 0.16				
Merlot (red)	Skins	33.3 ± 0.09	7.21 ± 0.04	8.80 ± 0.03	-		-	
	Seeds	124 ± 0.13	-	48.6 ± 0.34				
Smederevka (white)	Skins	29.9 ± 0.14	-	10.8 ± 0.05	-		-	
	Seeds	108 ± 0.05	-	49.4 ± 0.24				
Chardonnay (white)	Skins	8.71 ± 0.034	-	3.12 ± 0.12	-		-	
	Seeds	190 ± 0.20	-	69.6 ± 0.1				
Pinot Noir (red)	Skins	45.05 ± 0.85	1.21 ± 0.10	4.41 ± 0.12	3.31 ± 0.15	75.77 ± 1.12	87.61 ± 1.25	[64]
	Seeds	111.22 ± 1.28	-	51.50 ± 0.30	170.45 ± 2.52	579.33 ± 4.15	2203.51 ± 10.25	
Marselan (red)	Skins	56.17 ± 0.41	3.94 ± 0.15	6.70 ± 0.16	4.52 ± 0.14	89.74 ± 0.78	109.31 ± 1.01	
	Seeds	103.24 ± 1.11	0.062 ± 0.01	40.05 ± 0.18	152.18 ± 2.05	597.23 ± 4.12	2273.92 ± 12.32	
Cabernet Sauvignon (red)	Skins	42.32 ± 0.32	3.34 ± 0.12	6.45 ± 0.12	3.65 ± 0.13	81.23 ± 0.73	99.05 ± 0.88	
	Seeds	88.22 ± 0.72	0.05 ± 0.02	45.95 ± 0.14	157.22 ± 2.10	435.25 ± 3.3	2246.23 ± 11,337.9	
Tamyanka (white)	Skins	36.28 ± 0.29	0.015 ± 0.08	2.64 ± 0.11	1.23 ± 0.10	14.22 ± 0.18	58.23 ± 0.41	
	Seeds	79.06 ± 0.65	-	40.05 ± 0.18	31.44 ± 0.23	245.60 ± 3.23	1907.24 ± 9.56	
Touriga Naciona (red)	Skins	35.5 ± 1.8	9.3 ± 1.1	-	-	0.73 ± 0.04 **	33.2 ± 2.0	[65]
	Seeds	261.3 ± 7.0	-			0.09 ± 0.01 **	185.2 ± 5.9	
Preto Martinho (red)	Skins	360.2 ± 2.5	65.8 ± 8.1	-	-	0.15 ± 0.01 **	80.6 ± 3.1	
	Seeds	363.0 ± 0.5	-			0.05 ± 0.002 **	206.3 ± 7.7	

TPC—mg of gallic acid equivalents/g of extract; TA—mg of cyanidin-3 glucoside equivalents/g of extract; TF—mg of quercetin equivalents/g of extract; PC—mg of (+)-catechin equivalents/g of extract; DPPT—µM of Trolox equivalents/g of extract; ** EC_50_, mg/mL—extract concentration providing 50% of radical scavenging activity; ABTS—µM of Trolox equivalents/g of extract; * mg of ascorbic acid equivalents/g of extract.

One of the most important indicators of grape extracts is their antimicrobial activity. This indicator should be investigated, especially if the extract is intended to be used as an antimicrobial additive in foods or to obtain food packaging with antimicrobial action. There is a large number of publications proving the antimicrobial properties of grape seed and skin extracts against a number of pathogenic microorganisms [74,75,76]. There are many assumptions about the mechanism of antimicrobial action of polyphenols. It has been suggested that the antimicrobial effect of polyphenols may be due to the formation of complexes with polysaccharides and proteins [77], whereby extracellular or cell-bound enzymes are inhibited, for example, membrane transport proteins. Also, polyphenols are believed to bind metal ions and thereby inhibit bacterial growth [78]. Procyanidin polymers have more metal ion chelating sites, resulting in the greater inhibition of cell growth. The antimicrobial properties of grape extracts are studied in two ways, namely by the agar diffusion method and by determining their minimum inhibitory concentration against different microorganisms [79,80]. Table 3 shows the minimum inhibitory concentrations of grape skin and seed extracts of some grape varieties, which were published by some authors.

The results presented in Table 3 regarding the minimum inhibitory concentration of the extracts against a number of microorganisms show that the antimicrobial properties of grape seed extracts are more effective compared to those of grape skins. These results fully correlate with the richer content of phenolic compounds in grape seed extract. It is obvious that different bacterial species show different selectivity to the presented extracts, and this has been confirmed by many authors [74,75,76,79,81,82,83,84,85,86]. Moreover, in the presented data, it can be seen that the extracts are more effective against Gram-positive bacteria than against Gram-negative bacteria. The reason for this is the negatively charged liposaccharides located on the surfaces of the cell walls and an efflux pump that Gram-negative microorganisms possess [83]. The antimicrobial activity depends on the structure of phenolic compounds in grape extracts. Some authors indicate that the number of hydroxyl groups might dictate the inhibitory effect of each phenolic compound [87,88]. Others note that extracts with rich contents of catechin, epicatechin, and dimeric procyanidins have high antimicrobial capacity [89,90,91,92]. It has been indicated that the degree of polymerization of phenolic compounds also positively affects their antimicrobial capacity [93]. All of these compounds are present in grape seed and grape skin extracts and are more abundant in seed extracts.

**Table 3:** Minimum inhibitory concentrations (mg/mL) of grape skin and seed extracts of different grape varieties against some pathogenic microorganisms.

Grape Varieties	Extract Source	Microorganisms						References
		*Staphylococcus aureus*	*Staphylococcus epidermidis*	*Bacillus cereus*	*Listeria monocytogenes*	*Escherichia Coli*	*Klebsiella pneumoniae*	
Pinot Noir	seed	0.12	-	0.25	-	0.50	-	[64]
Marselan	seed	0.25	-	0.37	-	0.50	-	
Cabernet Sauvingnon	seed	0.37	-	0.37	-	0.75		
Touriga Nacional	seed	0.05	0.01	0.01	0.10	-	0.10	[65]
Touriga Nacional	skin	-	0.01	0.100	0.05	-	0.05	
Preto Martinho	seed	0.010	0.025	0.050	0.01	-	-	
Preto Martinho	skin	0.075	0.010	-	0.050	-	0.10	
Red Grape	seed	35	-	20	-	225	-	[82]
Pinot Noir	seed	0.78	-	-	-	25	-	[83]
Black Grape	seed	0.02	-	0.05	-	0.15	-	[84]

The combination of all phenolic compounds contained in the extracts provides a complex with a synergistic antimicrobial effect [81]. Some authors indicated that the antimicrobial action of the extracts is stronger than that of individual phenolic compounds precisely due to the synergistic actions of all phenolic components in the extracts [8,81]. Several studies have demonstrated that phenolic extracts have a stronger influence on the growth of microorganisms than individual compounds [94,95]. Serra et al. investigated the antimicrobial activity of grape extract containing quercetin at a concentration of 20 mg/L and the same concentration of pure synthetic quercetin against *Bacillus cereus* [94]. The mixture totally inhibited the growth of these bacteria, but they continued to grow in the presence of individual quercetin. Puupponen-Pimia et al. compared the antibacterial activity of berry extracts and separate fractions of pure flavonoids (including anthocyanins) and phenolic acids [95]. They found that polyphenolic mixtures were more effective than pure individual compounds. There are many publications investigating the synergistic effect between a mixture of several phenolic compounds in terms of their antioxidant capacity. Skroza et al. investigated the potential synergistic effect of a binary system of resveratrol with other phenolic compounds (gallic acid, caffeic acid, catechin, and quercetin) [96]. They found that there is synergy between catechin and resveratrol, caffeic acid and resveratrol, and gallic acid and resveratrol. Iacopini et al. investigated the antioxidant capacity of a mixture of five phenolic compounds [97]. The results indicate possible synergy between quercetin, rutin, and resveratrol towards authentic peroxynitrite ONOO−. Other authors presented that the two combinations: quercetin, gallic acid, and caffeic acid; and quercetin, gallic acid, and rutin have high synergistic effects [98]. It has been established that a synergistic effect not only occurs when polyphenols are complex mixtures, but also when they act simultaneously with an antibiotic or therapeutic drug. In some cases, subinhibitory concentrations of plant polyphenols can restore antibiotic sensitivity to bacteria that have previously acquired resistance [99]. Other authors suggest strengthening the antimicrobial effect of the extracts by adding an individual strong antioxidant to the grape extracts to obtain an “enriched extract” [100]. There are many questions to clarify in this direction, but the most important factor is that grape extracts have good antioxidant and antimicrobial capacities and can be successfully applied as antimicrobial additives in foods or included in the composition of food packaging.

## 4. Antimicrobial Food Packaging from Synthetic Polymers and Grape Seed and Skin Extracts

Food packaging is an important element in preserving food quality during the period from packaging to consumption [101,102]. Synthetic and natural polymers are used to produce film packaging. There is an increasing trend to obtain packaging with antimicrobial properties, as food spoilage due to microbial growth is one of the major problems faced by food manufacturers. Microbial growth in foods changes their quality and leads to an increased risk of disease. To reduce microbial contamination, synthetic chemical and natural antimicrobial agents are used, which are included directly in foods or in their packaging. It is known that added synthetic antimicrobial agents in foods exhibit cytotoxic effects [103,104], and therefore, natural plant agents have been increasingly used in recent years. The application of natural antimicrobial agents in packaging is a promising trend, as part of the antimicrobial agent migrates into the food and another part remains in the packaging and prevents the penetration of microbial contaminants from the external environment into the packaged food. These advantages have led to a new direction in the field of packaging materials—the use of antimicrobial packaging and the creation of new innovative packaging technologies.

For the preparation of packaging materials, several types of synthetic polymers are still widely used—polypropylene, polyvinyl chloride, polyethylene, and polyethylene terephthalate. The reason for this is their low cost, simple technologies, and high productivity. Their low permeability to oxygen and moisture makes them very suitable for the production of food packaging. They have very good barrier properties that minimize the penetration of external contaminants into packaged food products [105]. Synthetic polymers are known to be non-biodegradable, and there is a possibility of toxins migrating into food [106]. The migration of toxins leads to poor food quality and danger to the health of consumers. On the other hand, the non-biodegradability of synthetic polymers is a significant drawback and leads to environmental pollution [3,4]. Over the past few decades, the disposal of plastic waste, including food packaging, has led to dangerous consequences for the environment. Isotactic polypropylene is one of the most widely used synthetic polymers for food packaging. It has a low cost and very good rheological behavior, it is suitable for making polymer film, it is transparent, and it has good mechanical strength, durability, and a high heat deformation temperature [105]. In addition, it is recyclable, and its residues can be processed. Regardless of the listed advantages, the non-biodegradability of this synthetic polymer leads to great damage to the environment. In recent years, scientific research has been actively published, and innovative technologies have been developed for obtaining antimicrobial packaging based on both non-biodegradable synthetic polymers and biodegradable biopolymers [107]. Antimicrobial packaging is divided into two types. In the first type, there is direct contact between the antimicrobial surface of the package and the canned food, and the active agents can migrate into the food. In the second type of packaging, the antimicrobial surface is not in direct contact with the food [108,109,110,111]. These are high-barrier packages in which a mixture of gasses, such as CO_2_, H_2_, and O_2_, in a certain ratio are introduced to inhibit the growth of microorganisms in packaged foods [112]. In this review, we pay attention to the first type of antimicrobial packaging.

There are currently two major directions in the research and development of antimicrobial packaging technologies [3,113]. In the first direction, research is being conducted to modify non-biodegradable synthetic polymers and create packaging with better antimicrobial and antioxidant activities and better mechanical and barrier properties. This direction can easily be implemented because it does not require radical changes in production lines. But the main drawback of these packages remains, namely their inability to biodegrade and their tendency to lead to dangerous consequences for the environment. In the second direction, research is being conducted to obtain antimicrobial biodegradable packaging. The aim is to replace non-biodegradable polymers with biodegradable ones. This direction is very promising and environmentally friendly, but it is expensive and requires a significant change in existing technologies and production lines. The real confirmation of this direction will require a lot of funds and time, but its benefits in the field of health and environmental protection are very convincing. The development of new ecological and antimicrobial packaging technologies will emerge as an important and urgent task in the future.

Antimicrobial packaging is made in the form of films. First, a composite mixture is obtained by mixing and homogenizing a synthetic polymer or biopolymer with a bioactive component and then forming the film. Depending on the type of polymer and its properties, the films are formed by dry method or wet method [114,115]. The dry method does not require solvents. The films are formed by melt casting, extrusion, and heat pressing of the composite mixture. In the wet method, the film-forming ingredients are dispersed in solvents. The only suitable solvents are water, ethanol, and their mixture, since the film-forming solution should be edible and biodegradable [114]. The resulting polymer solution is degassed and cast onto a flat surface, and the solvent is then removed to create the film. There is also a variant in which the active component is applied in the form of a layer on the surface of the polymer film [115]. Usually, this option is used for films of synthetic polymers since they are produced by the dry method, and the applied temperature can partially inactivate the active component that is included in the matrix.

Due to the difficult implementation of the second direction in practice, requiring the creation of new technologies, new production lines, and significant funds, research relating to the first direction, describing the preparation of antimicrobial packaging based on synthetic polymers are still being published [116]. The deposition or inclusion of antimicrobial active substances in non-biodegradable films of polyvinyl chloride, polyethylene terephthalate, polyethylene, or polypropylene allows these packages to reduce microbial contamination in foods and extend their shelf lives [117]. In this review, the antimicrobial agents included in polymer films are mainly considered. Plant extracts with high antioxidant capacity are used as natural antimicrobial agents. Extracts from grape seeds, grape skins, or grape pomace (a mixture of skins and seeds) are widely used. Incorporating these extracts into polymer food packaging allows polyphenols to migrate into the food and inhibit microbial growth. In addition, the slow release of these bioagents ensures that the packaging has long-lasting antimicrobial activity. A no less important advantage is that the utilization of waste grape pomace or the seeds and skins obtained from it contributes to more sustainable and ecologically clean industrial production and reduces the adverse impact on the environment. The polyphenols contained in these residues are extracted using water, ethanol, or a mixture of the two. Each of these three extracts has its own benefits. The content of polyphenols is the highest in grape seed extracts, and they have the greatest application potential [24]. Grape skin extracts have a lower phenolic content than grape seed and pomace extracts, but they have one major advantage, namely a high anthocyanin content, especially when using red grape varieties. Thanks to anthocyanins, these packages not only provide antimicrobial action, but they also change color depending on the pH of the environment and indicate the suitability of the food product from receipt to consumption. This type of packaging belongs to a new development regarding packaging materials, namely intelligent packaging materials, specifically to the subtype of indicator packaging materials, which are used to directly monitor the quality of packaged food [118]. These intelligent materials are described in detail in Section 6.

There are many publications on the preparation of antimicrobial packaging based on non-biodegradable synthetic polymers and grape seed and skin extracts [116,119,120]. This type of film has excellent mechanical and barrier properties. The addition of grape extracts gives the films antimicrobial properties and makes them eco-friendly. Synthetic polymer films are mainly produced by the dry method. Heat treatment during film formation leads to the partial degradation of the more unstable phenols in the extracts. Nevertheless, publications indicate that these films have antimicrobial activity against a number of microorganisms tested. Tong et al. included grape seed extract in poly-ε-caprolactone and polyethylene films [119]. Poly-ε-caprolactone films are produced by pressing. The addition of grape seed extract leads to an increase in the crystallinity of poly-ε-caprolactone. The film has excellent antimicrobial activity against *Pseudomonas aeruginosa*. Polyethylene films with grape seed extract were obtained in two ways—via co-extrusion and the solution-coating process. This type of packaging is used to package ground beef stored at 3 °C. It shows extremely good antimicrobial properties against *E. coli* IFO 3301, *S. aureus* IFO 3060, and *B. subtilis* IFO 12113.

Rabello reported on the preparation of antimicrobial packaging based on isotactic polypropylene [120]. The author indicated that the addition of grape seed extract to isotactic polypropylene reduced oxidative degradation and inhibited the growth of microbial cells. Vasquez-Armenta et al. reported that grape rachis extract has a preservative effect against *L. monocytogenes* [121]. Thanks to the included grape extract in the polypropylene film, the adhesion of bacteria to its surface is reduced and the food quality is preserved.

Other authors have used grape pomace extracts (a mixture of skins and seeds) and incorporated them into polypropylene film [15]. The resulting bactericidal isotactic polypropylene (PP) film has low permeability and good mechanical properties. Thus, the modified packaging has the potential to function as an active and safer polymeric food packaging. Antibacterial activity tests show that the addition of grape extract leads to an increase in PP bactericidal activity against *E. coli* by more than 30% and against the bacterium *B. Subtilis* by more than 50%. The amounts of grape extract included in the PP film were 0.05 and 0.1 wt %. Authors have described that the polyphenols were found to undergo partial degradation during the heat treatment of PP. However, some polyphenolic compounds remain stable and retain their antimicrobial ability in the PP matrix.

Regardless of the stated good results obtained with the use of synthetic polymer packaging, their non-biodegradability limits their application. More than 90% of residual waste in landfills is due to food polymer packaging, and the difficulty in degrading them is a significant problem.

## 5. Antimicrobial Food Packaging made from Biopolymer and Grape Seed and Skin Extracts

In recent years, alternative packaging materials that are renewable, disposable, recyclable, and easily degradable have been actively sought [122]. Biodegradable polymers are suitable for this purpose. Thin packages that cover the food and are suitable for consumption are very preferred. Thus, a new promising direction arises in this field, namely the inclusion of active compounds in edible films of natural polymers [16]. The advantage of edible films is that they act as carriers of active substances, such as antioxidants, as antimicrobial agents, and as texture improvers [123]. In recent years, many publications about natural polymer films containing antimicrobial grape extracts have been presented [124,125,126]. The use of the extracts of grape pomace and grape seeds and skins (which are residual by-products of the winemaking process) as active agents makes these packaging technologies even more promising and economically profitable.

The following natural polymers are most commonly used to produce biodegradable films: cellulose, starch (natural and modified), chitosan, pectin, seaweed extracts (alginates, carrageenan, and agar), resins (acacia, tragacanth, and guar), polylactic acid, and pullulan [124]. Other significant natural sources are polysaccharides extracted from bacteria, fungi, and microalgae [127]. The main advantages of these packaging films are their biodegradability and non-toxicity and the possibility of not polluting the environment. But in order for these films to be used as packaging material for food, there also have requirements: a high permeability barrier, mechanical strength, and biochemical, physicochemical, and microbiological stability. No less important is the requirement that biopolymers be accessible and cheap [15]. Biopolymer films must be stable and non-exfoliating and be able to prevent gas or vapor exchange between the food and the atmosphere. Therefore, first of all, the following parameters of the film are examined according to established methods: their permeability to water vapor, mechanical strength, barrier properties, thickness, degradability, solubility, opacity, color, and thermal stability [128]. Secondly, as two of the most important parameters of antimicrobial packaging material, their antimicrobial and antioxidant capacities are investigated according to the same methods used for their determination in the original active extracts [128,129]. The migration of active agents from the packaging to the interior of the packaged food is also an important indicator when researching the packaging material. Despite the fact that the number of publications regarding this matter is small, the facts stated in these studies prove that there is a migration of the active agents to the food, which is an important advantage of antimicrobial packaging [130].

One of the most widely used polymers is chitosan. Chitosan films and coatings have been extensively studied since they are renewable, biocompatible, biodegradable, and non-toxic. They have been found to have antimicrobial properties without the addition of active antimicrobial agents. There are quite a few publications on the preparation of antimicrobial packaging films from chitosan alone or chitosan in a mixture with other polymers [131,132,133]. Chang et al. obtained an antimicrobial film by adding chitosan to polylactic acid (PLA) [134]. This film has an inhibitory effect of over 95% against *E. coli*, *Pseudomonas fluorescens*, and *S. aureus*. It is applied to package fish filets, which are then stored at 4 °C. Significant reductions in the microbe counts (i.e., mesophiles, psychrophiles, coliforms, *Pseudomonas*, *Aeromonas*, and Vibrio) were observed during the storage of fish filets, and the shelf life was prolonged to at least nine days. The activity of antimicrobial food packaging made from the biopolymers presented in this review against different microorganisms is shown in Table 4.

Although chitosan has antibacterial properties, in many publications, researchers add other active agents to it in order to improve its antioxidant and antimicrobial properties. They mainly seek a solution for determining the optimal amount of added active agent to the film. Increasing the concentration of the antimicrobial agent increases the antioxidant and antimicrobial properties of the film but reduces the mechanical strength and its barrier properties, so it is important to investigate these correlations. The type of solvent also affects both the extracted polyphenols and the mechanical properties of the film. Ferreira et al. investigated the effectiveness of adding grape pomace extracts to chitosan [135]. They used different extracts: water extracts (mainly polysaccharides), n-hexane extract (oil), and chloroform extract (wax). Chitosan films containing aqueous extract from grape pomace were found to be highly hydrophilic and silky. They retained their solubility in water and their mechanical strength. Moreover, these films showed a higher antioxidant capacity. The inclusion of chloroform grape pomace extract improves the flexibility of the films and their antioxidant properties and does not change their solubility. The results show that chitosan films containing the mentioned extracts have very good antimicrobial properties and can improve the shelf life of foods, but the films obtained with an aqueous extract are the most suitable as packaging materials because they are safe for humans.

The largest number of publications concerns the inclusion of grape seed extracts in a chitosan film. This is completely understandable considering the high content of polyphenols in these extracts. Shahbazi investigated the application of grape seed extract (1% *w*/*v*) in chitosan films [136]. He further investigated a combination of grape seed extract and *Zataria multiflora* essential oil from the *Ziziphora clinopodioides* plant. When comparing the results of the two types of films, it was found that they show very good antibacterial properties due to their high phenolic content. The authors Sogut and Seydim applied higher concentrations (5, 10, and 15 wt %) of grape seed extract (GSE) to a chitosan film [137]. Increasing the concentration of GSE lowers the transmittance and transparency of the film and increases the antioxidant properties of the film. The authors indicated that chitosan films containing grape seed extracts inhibited *E. coli*, *L. monocytogenes*, *S. aureus*, and *P. aeruginosa*. These films were found to inhibit the oxidation of chicken breast filets during refrigerated storage and showed a longer shelf life of vacuum-packed food under refrigerated conditions. Moreover, chitosan films containing 15% of grape extracts inhibited total mesophilic aerobic bacteria (TMAB) and coliforms in chicken breast filets. These results clearly show the potential of chitosan films with incorporated grape seed extracts for real-life applications as food packaging materials in the food industry. The approach used by Alves et al. to include grape seed extract in a chitosan film is interesting [138]. They applied two types of extracts in the form of microcapsules—grape seed extract and carvacrol (a phenolic monoterpenoid). The use of microcapsules allows the antioxidant capacity of the extracts to be preserved for a longer time. The mechanical and physico-chemical properties of the obtained film were investigated. Modified chitosan films reduce the growth of mesophilic and psychrophilic bacteria and *Pseudomonas* spp. These films are applied to salmon packaging. An increase in the shelf life of chilled salmon by up to 4–7 days of storage due to the antimicrobial effect of natural agents was found.

Grape seed extracts are known to have antifungal, antiviral, and antibacterial effects. Generally, most publications on antimicrobial packaging mainly examine their antibacterial properties. Amankwaah et al. not only studied the antibacterial properties, but also the antiviral properties of a chitosan film containing grape seed extract [139]. The resulting films were tested for the inactivation of murine norovirus (MNV-1), a surrogate for human norovirus. Additionally, their antimicrobial efficacy against *L. innocua* and *E. coli K12* was investigated. Chitosan films containing antimicrobial agents have been shown to have very good virucidal activity against MNV-1, *L. innocua*, and *E. coli K12* and can be successfully used to control food contamination by viruses and foodborne microbes.

There are publications on the preparation of antimicrobial packaging from other biopolymers as well—including pullulan, polylactic acid, alginate, and cellulose. Gomez obtained antimicrobial films from pullulan and polylactic acid (PLA) by incorporating grape seed extract and grapefruit seed extract with concentrations 1 and 5% [140]. It was indicated that the higher concentration of the extract reduced the mechanical properties of the films, while the antimicrobial activity of the films was the same at both concentrations of the extract. The modified films with extracts were found to have a very good inhibitory effect against the foodborne pathogens *L. monocytogenes*, *E. coli* O26, and *E. coli* O157:H7 and serovars Salmonella Infantis and Seftenberg.

Pažarauskaite et al. obtained interesting alginate films (2% *w*/*w*) by incorporating different concentrations of citric acid (5–20% *w*/*w*) and aqueous grape seed extract [141]. The polymer film was obtained by the solvent evaporation method. The role of the added citric acid is to crosslink the alginate and improve the mechanical properties of the film. Adding citric acid (up to 10%) results in a 33% increase in the film’s tensile strength and a 34% reduction in water vapor transmission. The resulting films have high antimicrobial activity against *E. coli* and *S. aureus*.

Cellulose and starch are also suitable and affordable biodegradable polymers. Xu et al. obtained starch nanocomposite films by incorporating grape pomace extract (GPE) and cellulose nanocrystal (CNC) [142]. The incorporation of CNC and GPE significantly increased the thickness, mechanical properties, and opacity of the films. GPE affects the brightness and color of films. CNC has a great influence on reducing the water vapor permeability of the film. The migration of phenolic compounds from packaging to food has been proven. The studied films with GPE and CNC showed a stronger inhibitory effect against *S. aureus* ATCC 29213 compared to *L. monocytogenes* ATCC 7644. Corrales et al. incorporated GSE into a pea starch film [130]. They demonstrated that 1% GSE reduced the growth of the bacterium *Brochothrix thermosphacta* that infected pork loins stored at 4 °C.

In the scientific literature, there are publications on the preparation of packaging films with less common biodegradable polymers. Complex compositions are often used in order to obtain films with good mechanical properties. Saurabh et al. developed a biodegradable active film based on guar gum (GG) to which tween-80 is added as an emulsifier, nanoclay is added as a reinforcement, beeswax is added for hydrophobicity, glycerol is added as a plasticizer, and grape pomace extract is added as an active agent [143]. Active films have very good tensile strength and a low water vapor transmission rate. The films demonstrated significant antimicrobial activity. Another complex composition used to prepare antimicrobial films was proposed by Deng et al. [144]. The authors used a mixture of plant-based polysaccharides, low methoxyl pectin, sodium alginate or Ticafilm^®^, and polyethylene. The resulting films had very good mechanical properties, and the amount of phenols released from the packaging ranged from 80 to 96%. The films showed antibacterial activity against both *E. coli* and *L. innocua*. Priyadarshi et al. obtained pectin-pullulan film with grape seed extract [145]. The mechanical strength of the polymer matrix with grape seed extract was increased. The film showed some antimicrobial activity against *E. coli* and *L. moncytogenes*. Row and roasted peanuts were coated with the obtained film. The author described that they achieved a 75% reduction in the peroxide values when they used pectin-pullulan film.

**Table 4:** Antimicrobial activity of different antimicrobial food packaging.

Polymers	Antimicrobial Extract Source	Targeted Microorganism	References
Poly-ε-caprolactone	Grape seeds	*P. aeruginosa*	[119]
Polyethylene	Grape seeds	*E. coli* IFO 3301, *S. aureus* IFO 3060, and *B. subtilis* IFO 12113	
Isotactic polypropylene	Grape rachis	*L. monocytogenes*	[121]
Isotactic polypropylene	Grape pomace	*E. coli* and *B.subtilis*	[15]
Chitosan–polylactic acid	-	*E. coli*, *P. fluorescens*, and *S. aureus* (inhibits mesophiles, psychrophiles, coliforms, Aeromonas, and Vibrio in fish filet)	[134]
Chitosan	Grape seeds	*E. coli* and *S. aureus*	[135]
Chitosan	Grape seeds	*E. coli*, *L. monocytogenes*, *S. aureus*, and *P. aeruginosa*. (inhibits total aerobic mesophiles and coliforms in chicken filet)	[137]
Chitosan	Microcapsules of grape seed and carvacrol extracts	*Pseudomonas* spp. (inhibits microorganisms in refrigerated salmon)	[138]
Chitosan	Grape seeds	Murine norovirus (MNV-1), *Listeria innocua*, and *E. coli* K12	[139]
Pullulan and polylactic acid	Grape seeds	*L. monocytogenes*, Salmonella Infantis and Seftenberg, *E. coli* O26, and *E. coli* O157:H7	[140]
Alginate	Grape seeds	*E. coli* and *S. aureus*	[141]
Starch and cellulose	Grape pomace	*S. aureus* ATCC 29213 and *L. monocytogenes* ATCC 7644	[142]
Guar gum	Grape pomace	*E. coli*, *S. aureus*, *B. cereus*, And *Salmonella typhimurium*	[143]
Polysaccharides and pectin	Grape pomace	*E. coli* and *Listeria innocua*	[144]
Pea starch	Grape seeds	In vitro with pork loins infected with *Brochothrix thermosphacta*	[130]
Pectin/pullulan	Grape seeds	*E. coli* and *L. monocytogenes*	[145]

## 6. Intelligent Antimicrobial Food Packaging made from Biopolymers and Grape Skin Extracts

In recent years, a new type of innovative packaging has been developed—so-called smart packaging. These are active packages that can indicate various changes in the packaged food. Through these materials, the quality of food can be monitored during its storage. Anthocyanins are a very suitable active ingredient in smart packaging. As a type of flavonoid, they have antioxidant and antimicrobial effects. Also, they change color at different pH values as their chemical structure changes. Therefore, they can serve as a pH colorimetric indicator for monitoring the freshness and spoilage of foods caused by microbial development [146,147]. Many studies have been published on anthocyanin packaging films. There are reviews that discuss the sources of anthocyanins [148,149], the types of natural indicators [150], and the methods of obtaining smart films and their applications [151]. The types of polymers used to obtain the films are also indicated, emphasizing biopolymers [152]. These studies describe that the incorporation of anthocyanins into films not only helps to monitor the quality of food, but also extends the shelf life of food and improves the physical and functional properties of food packaging films. Red grape skins are a very good source of anthocyanins [153]. The total phenols in grape skin extract are about 35%, with the main part being anthocyanins. Grape skins are separated from grape pomace, which is a waste product of winemaking. Grape skin extract is obtained through conventional and green techniques [154]. The obtained skin extract can be applied as a pH indicator and antimicrobial agent in food packaging. Different polymers are used to obtain colored films, but in recent years, biopolymers have had great applications.

Kannampilly and Thangavel investigated the possibility of developing a pH-sensitive indicator film based on the biopolymer ĸ-carrageenan [155]. The film contains anthocyanins extracted from grape pomace in three different concentrations: 0.5%, 1%, and 2%. The films show very good sensitivity to pH changes. The most noticeable color change was observed with the indicator film with 2% anthocyanin.

Chi et al. reported that they obtained a smart film from ĸ-carrageenan, hydroxypropyl methylcellulose, and anthocyanin-rich grape skin powder [156]. The authors found that when using lower concentrations of the active agent, it is distributed evenly in the film. The film has very good mechanical properties. In an acidic environment, the color of the film is pink, and at pH 7, it becomes blue-green. In order to evaluate its potential capabilities, pork monitoring tests were conducted, and the high sensitivity of the resulting indicator film was proven.

Etxabide et al. investigated the incorporation of tannin extracts from grape seeds (SeedT) and from grape skins (SkinT) into gelatin films [157]. Tannin extract from grape seeds was found to have a phenolic content about 30 times higher and 10 times higher antioxidant inhibition than those of grape skins. Both extracts were indicated to show color changes with the increasing pH of their stock solutions. When added to the gelatin film, they change the color of the film, reduce its wettability, and allow for a higher absorption of UV light. It was found that about 20% tannin could migrate from the films into the food, resulting in 13% antioxidant inhibition. The authors concluded that SeedT film is suitable for active packaging because the color change is small, while the SkinT film is suitable as a pH-sensing packaging for chilled foodstuffs, such as seafood and meat products, whose pH increases to basic values as a result of spoilage (due to volatile amines). The resulting smart SkinT films change color from gray-purple to green-blue when the pH of the food changes to basic values, and they serve as indicators of food spoilage over time.

Kamer et al. extracted anthocyanins from a solid winery by-product (Vinasse) [158]. They received three different colorimetric indicator smart films—polyvinyl alcohol extract, gelatin extract, and polyvinyl alcohol–gelatin extract. The extracts were found to improve the flexibility of the films. The polyvinyl alcohol–anthocyanin extract film showed the best pH sensitivity. The application of all three films to monitor shrimp freshness was investigated.

Ma et al. incorporated *Vitis amurensis* skin extracts from white grapes into the tara gum/cellulose matrix [159]. The color of the resulting film changes when the medium has different pH values. Films have been successfully applied to monitor the freshness of fish products, and it has been shown that they can be used as visual indicators for food quality assurance. In another publication [160] based on the same biopolymer and skin extract from red grape, a colorimetric indicator was obtained. The film changes color from red at an acidic pH to light green at an alkaline pH. The resulting film is applied to evaluate the freshness of milk. The observed color change in the film during this test shows that it can be used as an indicator package for food and give information about its freshness and suitability for consumption. The review of publications on smart food packaging obtained on the basis of biopolymers and grape skin extract clearly outlines the good prospects for the development of these innovative packaging materials in the future. Through them, consumers can not only monitor the freshness of food products, but also extend their shelf lives.

## 7. Conclusions

The development of antimicrobial food packaging is a promising direction as it solves important problems faced by food industry manufacturers. The application of these packages in practice would help maintain the quality of foods and extend their shelf lives. The main trends in their production are the replacement of synthetic polymers with biodegradable polymers and the use of natural plant agents with high antioxidant and antimicrobial capacities. Grape seed and skin extracts are very suitable active agents and provide good antimicrobial properties to the packaging. They are waste products of winemaking, which makes them affordable and economically viable. As a result of the utilization of this side waste, environmental protection is also achieved. In this way, a sustainable and circular economy can be built, starting from an affordable by-product of winemaking and obtaining active agents with a high added value and valuable antimicrobial food packaging. The presented data pave the way for the design and production of antimicrobial food packaging based on biopolymers and active agents derived from a widely available waste product obtained from the wine production chain.

## Figures and Tables

**Table 1:** The phenolic compounds in grape seeds and skins.

Resource	Phenolic Compounds	References
seeds	Phenolic acids (gallic acid, ellagic acid, syringic acid); flavan-3-ols ((+)-catechin, (−)-epicatechin, procyanidins (B1, B2, B3, B4); proanthocyanidins)	[22,23,24,25,26,27,28]
skins	Phenolic acids (gallic acid, ellagic acid, syringic acid), flavan-3-ols ((+)-catechin, (−)-epicatechin), proanthocyanidins (B1, B2, B3, B4)), flavonols (myricetin, quercetin, kaempferol, trans-resveratrol), anthocyanins (malvidin, cyanidin, oenin, delphinidin)	[22,24,26,27]

## Data Availability

Not applicable.
